# Factors influencing primary care physicians’ prescribing behavior of anticoagulant therapy for the management of patients with non-valvular atrial fibrillation in Singapore: a qualitative research study

**DOI:** 10.1186/s12875-021-01453-5

**Published:** 2021-05-25

**Authors:** Shera Chaterji, Lay Geok Lian, Ting Yee Lee, Liwei Chua, Sabrina Yi-Mei Wee, Sui Ling Yap, Dhana Letchimy K, Ngiap Chuan Tan

**Affiliations:** 1 SingHealth Polyclinics, 167, Jalan Bukit Merah, Connection One, Tower 5, #15-10, Singapore, 150167 Singapore; 2 SingHealth-Duke NUS Family Medicine Academic Clinical Programme, Singapore, Singapore

**Keywords:** Anticoagulants, Prescribing, Primary care physicians, Atrial fibrillation

## Abstract

**Background:**

Oral anticoagulant therapy use in patients with atrial fibrillation (AF) remains suboptimal in Singapore, despite the availability of both warfarin and non-vitamin K antagonist oral anticoagulants (NOACs). Primary care physicians’ (PCP) decision-making to initiate and select appropriate anticoagulant medication is pivotal in reducing complications among patients with AF. This study explored the factors influencing PCPs’ decision-making in anticoagulant initiation and anticoagulant switch for patients with non-valvular AF.

**Method:**

The study design is qualitative research based on the theoretical framework of the Generalist Wheel of Knowledge, Understanding and Inquiry. In-depth interviews or focus group discussions were conducted with 27 PCPs in general practice in urban Singapore. The audio-recordings were transcribed and coded to identify themes, which are framed according to the “clinician”, “patient”, “medical condition and treatment” and “healthcare system and policy” domains.

**Results:**

Personal training and experience with anticoagulant therapy; understanding patient risk-stratification; AF detection during clinical practice; medication cost; clinical support services for anticoagulation monitoring and constraints in existing care model influenced PCPs in their anticoagulant prescription. PCPs preferred to seek guidance from cardiologists in managing patients with newly diagnosed AF and attempted to engage their patients in decision-making regarding anticoagulant therapy. Some PCPs perceived sub-specialized primary care clinics focusing on AF co-management with cardiologists as an ideal setting for initiation and maintenance of anticoagulant therapy.

**Conclusions:**

PCPs are influenced by multiple interrelated factors while making decisions on anticoagulant initiation and anticoagulant switch for patients with AF. Their proposed care model to address the barriers awaits feasibility and acceptance assessment in future research.

**Supplementary Information:**

The online version contains supplementary material available at 10.1186/s12875-021-01453-5.

## Background

Atrial fibrillation (AF) is the most common arrhythmia in clinical practice. It is associated with a five-fold increase in stroke [1]. Scoring systems such as CHA_2_DS_2_-VASc have allowed risk stratification of patients with AF and guide physicians on the use of anticoagulants in patients with higher risk of stroke [2]. Anticoagulants such as warfarin significantly reduce the risk of stroke in patients with AF [3]. In the past decade, non-vitamin K antagonist oral anticoagulants (NOACs) have been approved for the treatment of non-valvular AF. NOACs are at least as effective as warfarin in preventing ischemic stroke in patients with AF [4–6]. In addition, NOACs offer several advantages over warfarin, such as fewer drug interactions and without the need for international normalized ratio (INR) monitoring [7].

Despite the availability of both warfarin and NOACs, a significant proportion of patients with AF still do not receive contemporary guideline-recommended anticoagulant therapy [8]. In Singapore, studies have shown that many AF patients in the tertiary care setting with high stroke-risk either did not receive warfarin or did not achieve adequate INR control while on warfarin [9, 10]. NOACs could be the anticoagulant of choice in some of these patients, however, NOACs are significantly more expensive compared to warfarin [11].

Previous studies in USA, UK and Canada have identified physician self-reported comfort level, perceived clinical benefits and risks, patient convenience and preferences and drug cost as factors influencing physicians’ decision to use anticoagulants in AF management [12–14]. A qualitative systematic review of physicians’ warfarin prescription in AF alluded to the challenges in care coordination across primary and tertiary care interface [15]. This has implications on the AF management by primary care physicians (PCPs). Patients may be first identified with AF in primary care. They may also either decline or default follow up by specialists. PCPs’ perspectives specifically on NOACs become critical in the management of these patients with AF but little is known of clinicians’ views and perspectives of this class of medications [16].

In Singapore, there has been a move to strengthen the primary healthcare services where PCPs assume expanding roles in managing the ageing population in the community. To reduce the burden and cost of tertiary care, stable patients from hospitals are appropriately stepped down to primary care for further management. Thus, PCPs increasingly shoulder greater responsibilities in managing such patients, who are previously managed in hospitals. They include those with AF. PCPs, especially those working in public primary care clinics (polyclinics) are already managing a significant segment of the population with non-communicable diseases. These polyclinics have in-house laboratories to assess INR with immediate results. Invariably, these PCPs encounter patients with newly diagnosed AF and face the imperative to initiate anticoagulants or to switch anticoagulants between warfarin and NOACs for patients with unstable INR control [17].

PCPs’ decision-making about anticoagulant initiation and anticoagulant switch for AF is complex [15, 16] and has significant impact on patients in reducing their risk of complications. However, there is a dearth of studies looking at factors which influence anticoagulant prescription by PCPs in Singapore and Southeast Asia. Hence, this study aimed to explore the factors which influenced PCPs’ decision-making in initiating and switching anticoagulant therapy for the management of patients with AF in Singapore. Identifying and addressing these factors will enable PCPs to optimally manage patients with AF and ensure their safety in the community.

## Method

A qualitative descriptive research approach was adopted in this study [18].

### Study site

The study site was SingHealth Polyclinics-Bukit Merah. It is located in southern Singapore serving an estate with a significant proportion of older population. About 40% of the 800 daily attendances at this polyclinic comprise of patients aged 65 years and older.

### Participants

PCPs practicing in ambulatory primary care settings such as polyclinics and private general practitioner (GP) clinics were invited to participate in this study. The participants self-declared to be in active clinical practice and had managed patients with AF. PCPs who were exclusively practicing in community hospitals and tertiary care settings were excluded as they had access to different healthcare resources. Purposive sampling of the participants from the professional networks of the study team was carried out to maximize the range of views based on their different practice settings, training background, qualifications and experience in AF management.

### Recruitment procedure

Letters of invitation were sent out by the principal investigator to eligible participants to participate in either a focus group discussion (FGD) or an in-depth interview (IDI) between December 2019 and November 2020. The letter of invitation stated the reasons for doing the study and emphasized that participation was voluntary. Written informed consent was taken prior to each FGD or IDI. FGDs were arranged for a mixed group of PCPs comprising medical officers, resident physicians and family physicians from a single polyclinic to allow exchange of ideas and examine views contextualized to the same practice setting. In-depth interviews (IDIs) were conducted for a medical officer and several senior PCPs to bring up issues related to different clusters of public polyclinics. IDIs and one FGD were arranged for private GPs and a locum doctor to identify unique challenges due their practice setting.

### Theoretical framework

A review of existing theoretical models on prescribing decisions showed that they mainly examined the relationship between marketing efforts and physician prescribing [19]. The Generalist Wheel of Knowledge, Understanding and Inquiry was eventually selected and adopted as the theoretical framework in this study for its contextualization to general and primary care practice [20]. The framework encompasses the “clinician”, “disease”, “patient” and “healthcare system & policy” domains and their inter-domain relationships. It enables the investigators to examine the PCPs’ prescribing behavior under the “clinician” domain in relations to their personal attributes, their understanding of AF and mastery of its treatment, their interactions with patients with AF when such therapy is indicated and the influences by the structure and processes in the local healthcare system and policies. “Integration” at the center of the framework allows a succinct and relational presentation of the themes across the domains.

### Topic guide

The semi-structured topic guide (Additional file 1) included questions about PCPs’ management of patients with AF, their experience with warfarin and NOACs, their clinical practice considerations, interactions with patients while choosing anticoagulants and opinions of using a patient decision-aid. These broad, open-ended questions allowed participants to cover the four major domains of the Generalist Wheel theoretical framework.

### Composition and profile of the study team

The study team comprised of 5 PCPs, an advanced practitioner nurse and 2 pharmacists practicing in SingHealth Polyclinics, a public primary care institution in Singapore. All members of the study team are involved in the care of patients and have special interest in improving anticoagulation therapy in AF.

### Data collection

Each FGD or IDI lasted about 30 to 40 mins. The moderator for both FGDs and IDIs was SC, a female PCP, with more than 5 years of clinical experience in family medicine and Master of Medicine qualification in family medicine. Another co-investigator, either LGL, DLK, SLY, SY-MW or NCT assisted SC during some of the interviews to take field notes. PCPs who worked at the study site and participated in the study were interviewed at the study site itself, in a quiet room of SingHealth Polyclinics-Bukit Merah. One interview with a GP was conducted at his clinic, for participant convenience. Subsequent interviews were affected by the restrictions on physical meetings imposed during the Covid-19 pandemic. After Institutional Review Board endorsement, these interviews were conducted with the remaining participants via the teleconferencing platform, Zoom.

Each participant was assigned a study identification number and transcripts were therefore de-identified. Before each session, participants completed a standardised questionnaire to record their demographics, practice setting, qualifications and experience. The same set of questions from the topic guide was used in the IDIs and FGDs, although the sequence could vary. Probes, prompts and follow-on questions were used during the IDIs and FGDs to facilitate discussion.

### Coding

The IDIs and FGDs were audio-recorded, transcribed verbatim and audited. The rectified transcripts were coded by two investigators to derive a first coding frame independently. Meetings were held regularly to discuss, modify and generate a final coding frame for data analysis based on the research questions and emerging themes. The final coding frame was subsequently applied to the remaining transcripts. Any discrepancies in coding were resolved after discussions with a third investigator. Representative quotes were selected after mutual agreement among the investigators to illustrate the study findings.

### Data analysis

The codes were used to identify emerging themes, which were then categorized according to the four key domains in the Generalist Wheel theoretical framework. The codes were also grouped under “relationship”, “information mastery” and “prioritization” at the clinician-patient, clinician-disease and disease-system interfaces respectively.

## Results

A total of 35 PCPs were invited to participate in this study, of which 8 of them declined to take part due to their busy schedules. 9 IDIs and 4 FGDs were conducted with 27 participants until saturation was reached.

The demographic characteristics and practice profiles of the 27 PCPs are shown in Table 1.

**Table 1:** Characteristics of participating PCPs

**Characteristic**	*n*
**Gender**	
Male	10
Female	17
**Age (in years)**	
Age ≤ 35	7
Age > 35—50	17
Age > 50	3
**Highest family medicine qualification**	
Bachelor of Medicine, Bachelor of Surgery (MBBS)	5
Doctor of Medicine (MD)	1
Graduate Diploma in Family Medicine (GDFM)^a^	6
Master of Medicine in Family Medicine (MMed)^a^	11
Fellowship (FCFPS)^a^	4
**Clinical practice setting**	
Polyclinic	21
General practitioner clinic	5
Locum	1
**Years of practice**	
< 10	9
10—19	13
> 19	5

^a^postgraduate qualifications in family medicine

**Fig. 1:**
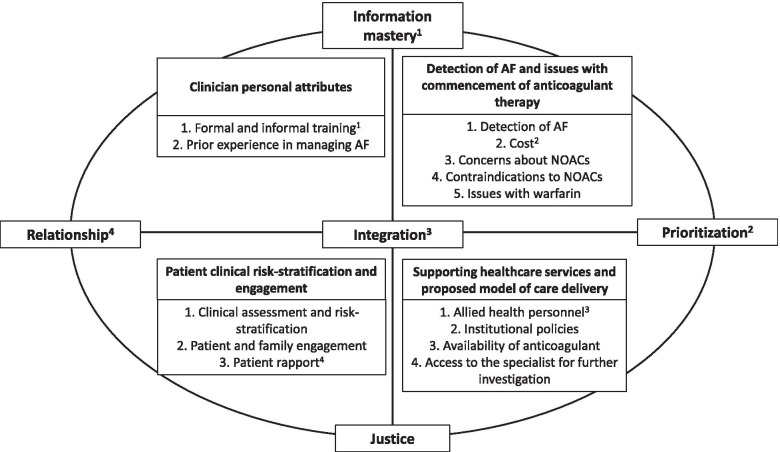
Factors influencing primary care physicians’ prescribing behavior of anticoagulant therapy for atrial fibrillation. Legend: Factors which influence anticoagulant therapy use by primary care physicians in atrial fibrillation presented according to the Generalist Wheel theoretical framework. Formal and informal training^1^, cost^2^, allied health personnel^3^ and patient rapport^4^ may also be categorized under information mastery^1^, prioritization^2^, integration^3^ and relationship^4^, respectively

The findings are summarized and presented in Fig. 1 according to the Generalist Wheel theoretical framework, focusing on the clinician domain and its interface with the “patient”, the “disease and treatment” and the “healthcare system and policy” domains.

### Clinician personal attributes

#### Prior experience in managing atrial fibrillation

Many PCPs, including those with postgraduate training, lacked personal experience in managing AF, which led to their uncertainty in anticoagulant therapy.

“*Whenever the primary care doctors have not done it very much, there’s some hesitancy to it because we are not too sure what to do and we don’t have that kind of experience behind us.”* P3, polyclinic PCP with family medicine postgraduate qualification.

*“I do start on Aspirin, but for anticoagulants, I think the main factor that I won’t start is just I am not so comfortable with it yet.”* P10, polyclinic PCP with family medicine postgraduate qualification.

Some PCPs highlighted the clinical challenges in picking up mitral stenosis, a condition which is a contraindication for NOACs. A few PCPs indicated that continuing medical education (CME) would help build their confidence in switching anticoagulants.

*“I don’t think my clinical skills are so good in picking up mitral stenosis; a diastolic murmur. So, I’m not confident…I mean for novel oral anticoagulants.*” P19, polyclinic PCP.

*“if you ask me to start anticoagulants, I’m not so comfortable in starting, but if I’m asked to follow up on a patient who is on anticoagulants, or having to switch the patient from warfarin to NOAC, I think with CME and teaching, for me, I think I’m still okay with doing that.”* P15, polyclinic PCP.

#### Formal and informal training

PCPs attributed their higher confidence in anticoagulant initiation and switch to specialized training courses and on-the-job learning from experienced senior physicians.

“*I think that postgraduate training or specialized family physician training courses would empower primary care doctors to accept and increasingly perform the role of initiation of anticoagulation.”* P25, GP.

*“I feel that training is also helpful in the sense that if a senior is starting a patient on NOAC and calls one or two colleagues to watch the consult, just for 10 min and see how they go about making that decision”* P24, polyclinic PCP.

### Patient clinical risk-stratification and engagement

#### Clinical assessment and risk-stratification

The patient profile, co-morbid conditions and demographics were taken into consideration by PCPs while making decisions about anticoagulants. PCPs relied on the CHA_2_DS_2_-VASc score and the HASBLED score to calculate the risk of stroke and bleeding, which also influenced their decision-making in initiating anticoagulants.

*“I guess the current quality of life of the patient, … also the demography will also be of some importance. If the patient is extremely old, even if they are still cognitively intact, sometimes, the benefit may not outweigh the risk.”* P2, GP.

*“I think the CHA*_*2*_*DS*_*2*_*-VASc score is the most important factor. So, the risk of stroke would determine how much I want to push for the patient to be started. HASBLED score, I guess has some impact on my decision making, but we do know that HASBLED score is not really a contraindication for atrial fibrillation.”* P23, polyclinic PCP.

Some PCPs rely on medical calculators available on their institution’s intranet, or on the internet to compute these scores for their patients.

*“in our intranet, we have the CHA*_*2*_*DS*_*2*_*-VASc score. HASBLED score, I don’t think there is. So, I’ll usually calculate the stroke risk at least. HASBLED, I mean, seeing the patient profile you will…if you think is very high, then I’ll calculate also.”* P22, polyclinic PCP.

*“if I want to calculate the CHA*_*2*_*DS*_*2*_*-VASc score, the HASBLED score, usually I use MD calculator, you know, that website….Yeah, so, it’s basically what I can find online.”* P21, GP.

#### Patient and family engagement

Most PCPs sought to involve their patients in shared decision-making regarding anticoagulants. Some PCPs also engaged patient’s family members to help the patient in this decision-making process.

*“good to have a shared decision-making process, where the patient himself also participates in this decision to start the medication. Also, because the population may be changing, we also start to realize that the patient, they themselves want to be able to be given a choice to decide.”* P20, GP.

“*if it’s the typical elderly individual, when it comes to such major decisions…. I usually ask them to come together with one or two of their children….at least they can get some assurance that one, you know they are doing the right decisions two, you know, sometimes if they do not understand what the physician says, their family may be able to put it in simpler terms for them.”* P21, GP.

PCPs generally relied on verbal discussions to engage their patients in this decision-making regarding anticoagulants. They reported that they lacked resources to engage patients further in this regard.

“*Yeah, it’s mostly a verbal discussion, now that there’s internet separation, I have to use my handphone to google something and show…I mean the more educated patients, I actually show them something on the webpages on the phone.*” P8, polyclinic PCP.

*“we don’t have anything in the layman language which can tell the patient about the drugs…..we just use our knowledge to discuss with the patient, but there is nothing, no resource which we can use.”* P24, polyclinic PCP.

“*at present, the practice is still a discussion between warfarin and NOACs…..we often just zoom in on comparing the two and then telling the patient the pros and cons and usually we highlight some key salient features…. Sometimes we are very busy, we may just focus on the few key issues and not cover the entire appropriate breadth and might just zoom in on just the cost and the side effects and the comparison between the two*.” P25, GP.

A few PCPs reported that despite adequate explanation, some patients chose not to opt for anticoagulation. In these instances, while patient autonomy about this decision is respected; PCPs also ensure that these patients are aware of the risk of withholding anticoagulants.

*“people who refuse anticoagulation nowadays are quite old, they have got multiple co-morbidities. What they usually say when they come in is that if I get a stroke, I get a stroke. I’m not ready to take my anticoagulation. I’ll just live with it when it comes. I’m so old already, anyway. Those ones are very hard to convince, but I still try, anyway to tell them that they need it.”* P1, polyclinic PCP.

“*of course, patient autonomy is important. So, there is also still the factor of patient acceptability. So, there are still patients, who despite good explanation, choose not to be anticoagulated and accept the inherent risks.*” P25, GP.

#### Patient rapport

Some PCPs acknowledged that their rapport with the patient would enable them to convince patients to follow their recommendation regarding anticoagulants.

*“if the doctor has good rapport, I think there’s a higher chance that they can convince patients to go on, because the patient trusts you and your decision-making skills.”* P3, polyclinic PCP.

PCPs acknowledged that an important part of the decision-making process regarding anticoagulants would be contextualizing the risk of stroke versus bleeding-risk for their individual patient. While PCPs seek to involve patients in shared decision-making while prescribing anticoagulants, there is a lack of readily available resources in the local setting to engage patients in this regard. This issue needs to be addressed to adequately empower more PCPs in Singapore to initiate or switch anticoagulants for AF patients in the community.

### Identification of AF and issues on the commencement of anticoagulant therapy

#### Detection of AF

PCPs occasionally detect AF incidentally during physical examination. They would refer symptomatic patients with giddiness or breathlessness to the hospital Accident and Emergency (A&E) department for further management.

*“the first thing is whether it’s incidental finding or not, for example usually it’s incidental finding when we check blood pressure …. we found that there’s irregular pulse. So, the patient is otherwise well.*” P4, polyclinic PCP.

*“if the patient is unstable or symptomatic….and you find out it’s because of AF, I will refer to A&E department.”* P13, polyclinic PCP.

#### Cost

Almost all PCPs mentioned cost as a significant factor influencing their anticoagulant prescription for NOACs. Moreover, the cost of NOACs is substantially higher than warfarin as illustrated in the quote below from P2. However, some PCPs acknowledged that patients on warfarin also incurred additional costs for the periodic INR monitoring at the laboratories.

*“I would say cost is a very big factor ….in our clinic we are selling 1 tablet of NOAC at around $4 to $5. That would be approximately S$2000 a year, just for one medication. So, if they were to be taking warfarin, maybe it will be even less than S$100.”* P2, GP.

*“a big part would be money you see, because the NOACs tend to be more expensive. Warfarin tends to be cheaper, however, you know coming to do blood tests every 3-monthly might be quite costly as well.”* P21, GP.

#### Concerns about NOACs

PCPs had concerns about NOACs, such as the lack of monitoring and the lack of antidotes.

*“it takes 24 h for the drug to wear off and there is no real antidote, except for dabigatran …The second would be…we don’t really know how anticoagulated they really are…. because there is no way to measure”* P2, GP.

#### Contraindications to NOACs

While most PCPs were aware of contraindications to NOACs, such as renal impairment and liver disease, they were concerned about missing the diagnosis of mitral stenosis. Some of them would rely on an echocardiogram to identify mitral stenosis.

*“we do know that if they have any abnormal liver or kidney function, they shouldn’t be on certain medications like the NOACs.”* P20, GP.

*“I must admit that I myself have not converted anyone from warfarin to NOACs. The first things that I would be concerned with is to find out whether there has been a history of 2D Echo done. To see whether there is any significant mitral stenosis.”* P17, polyclinic PCP.

#### Issues with warfarin as the alternative

Multiple issues with warfarin such as labile INR, diet and drug interactions were raised by PCPs. These issues may trigger PCPs to switch to NOACs.

*“if the patient is taking a number of medications and there is drug interactions, and also, the patient doesn’t tend to come back regularly for follow up… In those cases, I may actually switch the patient to NOAC, if they don’t have any contraindications.”* P15, polyclinic PCP.

The higher cost of NOACs and concerns about the lack of monitoring of anticoagulation status with NOACs are barriers to the initiation of NOACs in primary care. Additionally, PCPs expressed concern about missing mitral stenosis, which is detectable via echocardiogram, an investigation which not many PCPs have access to. On the other hand, while warfarin has been available for a longer duration, there are numerous challenges with its’ use, which often prompts PCPs to consider switching to NOACs. Therefore, in order to enable more PCPs to initiate NOACs, or switch between warfarin and NOACs, modifiable factors such as cost of NOACs and PCPs’ access to tertiary investigations like echocardiograms should be addressed further.

### Supporting healthcare services and proposed model of care delivery

#### Allied health personnel

PCPs practising in public polyclinics suggested enlisting the services of the pharmacists in medication counselling and the Medical Social Workers (MSWs) in financial counselling. MSWs counsel patients on their eligibility for subsidies based on their socioeconomic background and render assistance to those who are unable to afford the more expensive NOACs.

*“I feel that the allied team will be very useful. I mean the pharmacists can help a lot in terms of counselling because we may not have that much time to counsel the patient and to elicit the patient’s other concerns”* P8, polyclinic PCP.

*“The other allied services that we can tag on is the social workers…. so that they can counsel the patient and find out whether the patient is eligible for different financial subsidies that are available, so that they can start on the medicine.”* P6, polyclinic PCP.

In contrast to patients seen in public polyclinics, patients seen in GP clinics do not have access to subsidies provided to reduce the cost of NOACs. Therefore, some GPs refer their patients who have issues with the cost of NOACs to the polyclinics where these patients may be eligible for subsidies.

*“often we’ll tell them to go to the hospital pharmacies to take it because, usually the mark up is different compared to the private pharmacy. However, there’s always this thing where I will have to discuss with the patient whether they will want to go to the polyclinic for subsidised management of their condition.”* P20, GP.

#### Institutional policies

PCPs are guided by their institutional policies regarding starting anticoagulants. They follow the instructions in the electronic doctor training manual provided by the institution for reference.

*“We still cannot start warfarin, even if we detect a new AF, because we don’t have the policy to manage them here, to reach the adequate INR targets…because it takes time to do this”* P6, polyclinic PCP.

#### Availability of anticoagulant

The PCPs who practice in private GP clinics reported that their prescriptions were influenced by available anticoagulants in the formulary or stock of their practice.

*“I work in a group practice, it also depends what my group carries, like we only have Xarelto, for example. Xarelto and like warfarin 5 mg or 3 mg. So, you just have to play around with whatever you have”* P21, GP.

#### Access to the specialists for further evaluation

Most PCPs would seek the guidance of the cardiologist in managing patients with newly diagnosed atrial fibrillation. This is usually in the form of a referral to the cardiology clinic.

*“because of the accessibility of tertiary care, specialist care in Singapore, usually upon diagnosis, I would prefer to refer to the tertiary care first, you see. Because sometimes you do need to find the underlying cause of the AF. Rule out things like whether it’s due to a heart problem. So, usually these tests can only be done in the tertiary setting and it can be done pretty fast, sometimes.”* P21, GP.

However, two PCPs interviewed for this study manage specialized AF clinics in their respective primary care practice in collaboration with cardiologists. They also have access to facilities to perform 2D Echocardiograms. This in turn empowered the PCPs to start anticoagulants in the primary care setting.

“*So, we have atrial fibrillation clinic, which is run in conjunction with the cardiologists. We have access to specialist opinion *via* phone call or message very, very readily if we need some advice. If not, then we have access to order 2D Echo and all these on our own, without referring directly. We can do all these things in the primary care setting and start anticoagulants”* P23, polyclinic PCP.

Allied health personnel in public polyclinics support PCPs in anticoagulant prescribing by counselling patients about anticoagulants. MSWs play an important role in determining whether patients are eligible for subsidies in polyclinics so that they can be started on NOACs. In contrast, GPs do not have access to allied health personnel and may also face limitations in prescribing anticoagulants depending on the availability of medications in their respective clinics. While most PCPs would seek cardiologist advice to manage patients with newly diagnosed AF, some PCPs already run AF clinics in collaboration with cardiologists, thereby empowering them with confidence and experience in prescribing anticoagulants for patients with AF.

## Discussion

This study has highlighted the complex interrelated factors which influence the PCPs’ prescribing behavior of anticoagulants in patients with AF. Clinician training and experience, clinical risk-stratification, patient engagement, support services and care model affect both their anticoagulant initiation and switch from warfarin to the NOACs, or vice versa. They were also influenced by medication specific factors such as the need for anticoagulation monitoring and cost to patients. As illustrated in Fig. 1, these factors span across the clinician, patient, disease and healthcare system domains.

NOACs have many advantages compared to warfarin, such as fewer drug and food interactions, no requirement for regular INR monitoring and lower risk of intracranial bleeding [7]. Therefore, in keeping with contemporary international guidelines, NOACs should be the preferred anticoagulant for stroke prevention in patients with non-valvular AF in Singapore [11, 21, 22]. Although PCPs are knowledgeable about the risk-scoring in atrial fibrillation and the contraindications to NOAC use, they highlighted concerns about lack of personal experience in managing AF, which contributed to their hesitancy to start anticoagulants. Despite these concerns, many PCPs in Singapore have already been continuing anticoagulants for their patients with AF, who may have been stepped down from tertiary care to the primary care setting. As Singapore moves to strengthen its primary healthcare services, PCPs will increasingly assume responsibility for initiating NOACs for patients with AF if their CHA_2_DS_2_-VASc score changes with time, or will need to consider switching anticoagulants for patients who have issues with warfarin. While the lack of confidence and experience among family physicians prescribing and controlling warfarin has been recognized in the past [15], these same issues continue to persist in the urban healthcare setting of Singapore despite the availability of the newer NOACs and their relative ease of use. It remains to be seen whether with time, as the use of NOACs becomes more commonplace, PCPs gain more experience and thus confidence, in initiating and switching to this category of anticoagulants.

In the local fee-for-service primary healthcare system, cost was a common theme raised by most PCPs as patients are required to pay for their consultation, laboratory investigations and medications. Government subsidies are available to reduce the healthcare expenditure of patients in the polyclinics and selected GP clinics. The costs of NOACs such as rivaroxaban and apixaban remain high for patients compared to warfarin [31], unless they are eligible for financial assistance after review by the MSW. Hence, the subsidies are dependent on the financial status of the patient. Notwithstanding the cost of NOACs, patients on warfarin also incur additional costs in laboratory INR monitoring and associated consultations. A study on the local PCPs’ prescribing behavior of the expensive long acting beta-2 agonist inhalers for patients with persistent asthma showed similar concern on cost when these medications were initially launched [24]. However, a subsequent related study revealed that expenditure declined over time due to reductions in complications and hospitalization [25]. The availability of generic brands of NOACs may become more accessible to patients with AF over time and cost-effectiveness of their use may become enhanced.

PCPs admit that patients themselves want to be involved in the decision-making process regarding anticoagulant therapy. The medical fraternity is increasingly recognizing the importance of personalized decision-making by patient themselves [15, 26]. However, there is a lack of locally available resources to engage patients on these discussions about anticoagulant therapy. Patients’ perspectives on these decisions about anticoagulants would be important in order to develop such resources and enhance the decision-making process, but this was not explored in our study. One such resource which is potentially useful in the decision-making process regarding AF is a patient decision aid (PDA), which has advantages including reducing decisional conflict, increasing patient knowledge, clarifying patients’ values and improving decisional quality [27]. However, few validated PDAs on AF anticoagulant therapy are accessible for clinical use [28]. The results of this study will be valuable in designing a PDA template, including listing the cost of the NOACs. Input from patients should be sought to culturally adapt such a PDA to the local context.

The two PCPs working in specialized AF clinics in primary care indicated that tele-collaboration with the cardiologists facilitated clinical decisions on anticoagulant therapy in AF. Other care models established in the UK, Netherlands and Spain have shown that cardiologists who serve in integrated primary care clinics effectively deliver oral anticoagulation to high-risk AF patients in the community [29–31]. In Singapore, a multidisciplinary collaborative team should be considered to strengthen the AF management in primary care. The team could include pharmacists and MSWs to co-manage patients with AF by providing medication and financial counselling respectively. The limited availability of anticoagulants in private GP clinics may be addressed by allowing patients to refill their prescriptions at polyclinics or public hospitals or through the setting up of centralized national pharmacy at convenient locations.

The results of this study highlight the multiple interrelated factors influencing PCPs in Singapore when they make decisions about initiating or switching anticoagulants for patients with AF in the community. It illustrates the multi-domain barriers faced by PCPs as they assume new roles in care delivery for patients with AF who were previously managed predominantly by specialists. The PCP-specialist collaborative tele-support care model proposed by some PCPs is a potential solution to the multitude of barriers but its effectiveness must be evaluated through robust health service research and implementation science.

The deployment of the Generalist Wheel framework is a strength in this study. It readily presents the readers a more comprehensive understanding of the complex inter-related issues influencing anticoagulant prescribing behavior specifically for the PCPs. With evolving information and emerging guidelines regarding anticoagulant use in AF, it is likely that their prescribing behavior will change with time. A different model of care will potentially accelerate the change, which ultimately should deliver evidence-based treatment to patients with AF.

The study has its limitations. The results could not be generalized to the anticoagulant prescribing behavior of the entire local PCPs, although the purposive recruitment ensured documentation of the views of PCPs in both public and private healthcare settings. The views of patients are equally critical as they are the recipients of the anticoagulant therapy. Future studies are needed to explore the patients’ decision-making in taking such medication and their acceptability to receive treatment in the new care model. The investigators plan to leverage on the perspectives of the PCPs to create a PDA on anticoagulants and to assess if it helps patients in their medical decisions to use the NOACs.

## Conclusion

PCPs are influenced by multiple interrelated factors while making decisions on anticoagulant initiation and anticoagulant switch for patients with AF. Some PCPs preferred to collaborate with cardiologists in managing patients with newly diagnosed AF. Their proposed PCP-specialist collaborative tele-support care model is a potential solution to optimize AF treatment in the community but such sub-specialized AF clinic in primary care awaits further assessment on its feasibility and acceptance by both PCPs and patients.

## Supplementary Information

**Additional file 1.** Topic Guide

## Data Availability

The datasets analysed during the study are available from the corresponding author on request.

## Abbreviations

AF: Atrial fibrillation
CME: Continuing medical education
FGD: Focus group discussion
GP: General Practitioner
IDI: In-depth interview
INR: International normalized ratio
MSW: Medical Social Worker
NOAC: Non-vitamin K antagonist oral anticoagulants
PCP: Primary care physician
PDA: Patient decision aid
