# Supplementary material for: Factors influencing primary care physicians’ prescribing behavior of anticoagulant therapy for the management of patients with non-valvular atrial fibrillation in Singapore: a qualitative research study
Source: BMC Fam Pract. 2021 May 25;22:101. doi: 10.1186/s12875-021-01453-5 (PMC8146184; doi:10.1186/s12875-021-01453-5)
Supplement: Supplementary file 1 — Additional file 1. Topic Guide [file 12875_2021_1453_MOESM1_ESM.docx]

**Factors influencing primary care physicians’ prescribing behavior of anticoagulant therapy for the management of patients with non-valvular atrial fibrillation in Singapore: A Qualitative Research Study**

Shera Chaterji^1^, Lay Geok Lian^1^, Lee Ting Yee^1^, Chua Liwei^1^, Wee Yi-Mei Sabrina^1,2^, Yap Sui Ling^1^, K Dhana Letchimy^1^, Ngiap Chuan Tan^1,2^

^1^ SingHealth Polyclinics, Singapore

^2^ SingHealth-Duke NUS Family Medicine Academic Clinical Programme, Singapore

**Appendix 1: Topic Guide**

| **Questions** | **Domains** |
| --- | --- |
| 1. Tell me how you manage patients with AF. | Clinician, disease and treatment, healthcare system and policy. |
| 2. Tell me more about your experience with warfarin. | Clinician, disease and treatment. |
| 3. Tell me more about your experience with NOACs. | Clinician, disease and treatment. |
| 4. What are your considerations while choosing anticoagulants in patients with AF? | Patient, disease and treatment, healthcare system and policy. |

*A separate question on the proposed patient decision-support aid on anticoagulant therapy for AF is presented in a separate publication.
